# B7 Costimulation Molecules Encoded by Replication-Defective, vhs-Deficient HSV-1 Improve Vaccine-Induced Protection against Corneal Disease

**DOI:** 10.1371/journal.pone.0022772

**Published:** 2011-08-03

**Authors:** Jane E. Schrimpf, Eleain M. Tu, Hong Wang, Yee M. Wong, Lynda A. Morrison

**Affiliations:** Department of Molecular Microbiology and Immunology, Saint Louis University School of Medicine, St. Louis, Missouri, United States of America; Southern Illinois University School of Medicine, United States of America

## Abstract

Herpes simplex virus 1 (HSV-1) causes herpes stromal keratitis (HSK), a sight-threatening disease of the cornea for which no vaccine exists. A replication-defective, HSV-1 prototype vaccine bearing deletions in the genes encoding ICP8 and the virion host shutoff (vhs) protein reduces HSV-1 replication and disease in a mouse model of HSK. Here we demonstrate that combining deletion of ICP8 and vhs with virus-based expression of B7 costimulation molecules created a vaccine strain that enhanced T cell responses to HSV-1 compared with the ICP8^−^vhs^−^ parental strain, and reduced the incidence of keratitis and acute infection of the nervous system after corneal challenge. Post-challenge T cell infiltration of the trigeminal ganglia and antigen-specific recall responses in local lymph nodes correlated with protection. Thus, B7 costimulation molecules expressed from the genome of a replication-defective, ICP8^−^vhs^−^ virus enhance vaccine efficacy by further reducing HSK.

## Introduction

Herpes simplex virus 1 (HSV-1) infections are ubiquitous in the population world-wide and in the United States, where seroprevalence is 65% by age 50 [1]. HSV-1 remains a frequent cause of eye infections, afflicting up to 500,000 persons each year in the United States [2], [3]. Periodic HSV-1 reactivations instigate recurrent infection of the cornea, resulting in immunopathologic damage and HSK. For some, corneal scarring leads to loss of vision; HSK is the second most common cause of non-traumatic corneal blindness [3]. Development of an effective vaccine against HSV-1 would help control or prevent this sight-threatening disease.

Effective control of HSV infection depends on the antiviral T cell response. Activation of naïve T cells requires three signals: T cell receptor engagement of the appropriate antigen/MHC molecule, interaction of CD28 with B7-1 and B7-2 costimulation molecules, and cytokines that drive T cell differentiation. Antiviral vaccines must elicit or provide these signals in order to induce strong cell-mediated immunity. Glycoprotein, peptide, or plasmid-based vaccines can decrease corneal shedding of HSV-1 and reduce the severity of HSK [4]–[7]. DNA vaccines provide antigen to T cells, and induce costimulation molecule expression due to inherent CpG motifs. Nevertheless, repeated vaccinations are usually required to achieve protection. Similarly, viral glycoproteins or peptide epitopes provide only antigen, so they require mixture with adjuvant to supply the “danger signals” necessary to elicit costimulation and cytokines. Vaccine preparations consisting of or encoding multiple glycoproteins are more potent than a single glycoprotein [8], indicating the benefits of a multivalent vaccine. Attenuated, replication-competent viruses as vaccines naturally stimulate responses to multiple epitopes and also supply the necessary danger signals by virtue of their similarity to wild-type virus infection. Neuroattenuated mutants of HSV-1 successfully reduce viral replication and HSV-mediated corneal disease in mice [9]–[11]. However, attenuated HSV-1 can still be amplified 10,000-fold in tissue culture [9], and can develop adventitious mutations [12], raising safety concerns about replication-competent agents as vaccines.

To address the needs for both safety and immunogenicity in a vaccine, replication-defective viruses have also been explored as mimetics of virus infection to prevent HSV-1 infection and eye disease [13], [14]. HSV-1 strains made replication-defective by disruption of the UL29 gene encoding ICP8, essential for viral DNA replication, have shown promise in a mouse model of corneal infection. A single immunization with ICP8^−^ virus reduces HSV-1 replication in the cornea after challenge, acute and latent infection of the trigeminal ganglia (TG), and incidence of HSK [14]. ICP8^−^ replication-defective HSV-1 induces T cell proliferative and cytolytic responses [14], [15]. CD8^+^ T cells appear to protect against immunopathologic damage to the cornea following HSV infection [16], [17], while CD4^+^ T cells reduce virus replication in the cornea and latent infection in the TG [16].

Despite these benefits, virus-encoded immunomodulators may diminish the strength of immune stimulation with an ICP8^−^ HSV-1. For example, the virion host shutoff (vhs) protein encoded by UL41 helps HSV evade both innate and adaptive immunity [18]–[21]. Indeed, deletion of vhs from an ICP8^−^ HSV-1 vaccine increases the virus' capacity to protect mice against replication, disease and latency after corneal challenge with HSV-1 [22]. Suboptimal immune stimulation with replication-defective virus may also occur if contact with professional antigen presenting cells (APCs) is limited. The increased severity of HSV infections in mice lacking B7-1 and B7-2 costimulation molecules (B7KO) testifies to the importance of costimulation in development of HSV-specific immunity [23]. We have previously demonstrated that vaccination with replication-defective HSV-2 encoding B7-1 or B7-2 from within the viral genome partially restores protective immune responses against HSV-2 to B7-1/B7-2^−/−^ (B7KO) mice [24]. B7-2-expressing, replication-defective HSV-2 also affords wild-type mice better protection against HSV-2 infection than does the parental replication-defective virus [25], even though wild-type mice express endogenous B7 molecules.

Thus, we had previously shown that deletion of vhs from a replication-defective HSV-1 improves its protective efficacy as a vaccine, and that addition of B7 coding capacity to a replication-defective HSV-2 improves its effectiveness as a vaccine. In the current study we constructed an HSV-1 mutant containing the vhs deletion and encoding B7 costimulation molecules (ICP8^−^vhs^−^B7^+^ HSV-1) to determine whether the effects of vhs deletion and B7 insertion would be additive and so create a more promising HSV-1 vaccine candidate.

## Materials and Methods

### Ethics statement

This study was carried out in strict accordance with the recommendations in the Guide for the Care and Use of Laboratory Animals of the National Institutes of Health. The protocol and study was approved by the Committee on the Care and Use of Animals of Saint Louis University (NIH assurance number A3225-01; Institutional protocol number 1136). All procedures were conducted in a manner to minimize suffering.

### Cells and viruses

The replication-defective mutant of HSV-1 KOS, Δ41Δ29 [22], has defects in expression of vhs and the essential gene product ICP8 due to insertion of a nonsense linker in the UL41 open reading frame (ORF) at amino acid position 238 [26] and disruption of the UL29 ORF due to insertion of a lacZ expression cassette, respectively. Δ41Δ29 was propagated in S2 cells, a Vero cell line stably expressing ICP8 [27]. Δ41Δ29 was further mutated to contain a murine B7-1 (CD80) or B7-2 (CD86) expression cassette. The CD80 and CD86 ORFs, cloned downstream of the HCMV immediate early enhancer/promoter in plasmids pBS(HCMV/B7-1) and pEH48(HCMV/B7-2) [24] were excised and inserted into a BglII site previously engineered 751 bp from the 5′ end of the thymidine kinase (tk) (UL23) ORF in plasmid p101086.7BglII (Dorne Yager and Don Coen, unpublished). These plasmids were cotransfected with full-length Δ41Δ29 DNA into S2 cells using nucleofection (Amaxa Biosystems), according to the manufacturer's protocol. To select B7-expressing recombinant viruses, S2 cells infected with virus progeny of the cotransfection were incubated in the presence of 100 µM acyclovir. Potential recombinant viruses capable of growing in the presence of acyclovir were grouped in pools. Fresh cells infected with each pool were screened by flow cytometry for expression of B7 molecules (see below). Isolates from positive pools were individually re-screened by flow cytometry and then plaque-purified to homogeneity. Insertion into the *tk* locus was confirmed by Southern blot analysis. The B7-1- and B7-2-expressing viruses were named Δ41Δ29B7-1 and Δ41Δ29B7-2, respectively. Viruses used for immunizations were produced free of cell debris by isolation from the supernatant of infected cell monolayers using high speed centrifugation as previously described [28]. HSV-1 strain microplaque (mP) [29] was propagated in Vero cells. Virus titers were determined on S2 or Vero cells by standard plaque assay [30].

### Mice

Female BALB/c mice (H-2^d^) were purchased from the National Cancer Institute. Female BALB.B mice (H-2^b^) were purchased from The Jackson Laboratories. Female B7KO mice [31], backcrossed onto a BALB/c background, were bred at Saint Louis University and housed in sterile microisolator cages. All mice were housed at Saint Louis University under specific-pathogen-free conditions and were used at 6 wk of age.

### Southern blot hybridization

Viral DNAs were purified from potential recombinant viruses using a Qiagen QIAamp DNA Mini Kit according to the manufacturer's instructions. One µg of each DNA sample was subjected to *EcoR*I restriction digestion, and fragments were separated on a 0.8% agarose gel. DNA fragments were transferred to Hybond-N+ nylon membrane (Amersham) by capillary diffusion and hybridized to a randomly primed, [^32^P]-labeled *Sac*I fragment of plasmid p101086.7 used as a probe. Images were obtained on X-ray film by autoradiography.

### Flow cytofluorometric analyses

S2 cells infected with potential recombinant plaque isolates were stained 24 hr later by addition of anti-B7-1 or B7-2-biotin (1∶150; PharMingen/Becton-Dickinson), followed by streptavidin-FITC (1∶150; Immunotech) and analyzed by flow cytometry on a FACSCalibur. For demonstration of B7 expression by Δ41Δ29B7-1 and Δ41Δ29B7-2, S2 cells were stained 24 hr after infection at moi of 5 by addition of anti-B7-1 or B7-2 biotin and streptavidin-FITC, and with anti-HSV-1 rabbit antiserum (1∶100; Dako) followed by goat anti-rabbit-phycoerythrin (PE) (1∶100; Vector Laboratories).

For intracellular cytokine staining of CD4^+^ T cells, groups of BALB.B mice were immunized subcutaneously (s.c.) in the hind flanks with 4×10^5^ pfu of virus or control supernatant. After 6 d draining lymph nodes were removed and single cell suspensions were made. Cells were cultured for 4 hr in the presence of phorbol myristate acetate (PMA; 50 ng/ml), calcium ionophore A23187 (CaI; 1 µg/ml), and GolgiStop (0.67 µl/ml; PharMingen). Cells were treated with Fc block, followed by anti-CD3-PerCP and anti-CD4-Pacific Blue, then were fixed and permeabilized using a cytostain kit (PharMingen), and stained with anti-IFNγ-PE. T cells were analyzed by flow cytometry using an LSRII (Becton Dickinson) and FloJo 8.0 software.

### ELISpot

Groups of BALB.B mice were immunized with 4×10^5^ pfu of the various vaccine strains or an equivalent amount of control supernatant suspended in 40 µl total vol of normal saline. For acute immune responses, paraaortic and inguinal lymph nodes were removed 6 d later and dilutions of 1×10^5^ to 1.5×10^4^ cells from individual mice were added per well in duplicate to Milliscreen-HA plates (Millipore) previously coated with antibody to IFNγ (BD Pharmingen). HSV-1 gB peptide 498–505 [32], [33] was added to the cultures at 0.2 µM. Alternatively, groups of BALB/c mice were immunized as above and paraaortic and inguinal lymph node cells were cultured at concentrations of 1×10^6^ to 1×10^5^ cells per well along with UV-inactivated HSV-1 virions (equivalent to 1×10^5^ pfu prior to inactivation). After incubation for 20 hr, plates were washed extensively to remove cells and captured IFNγ was detected using a biotinylated anti-IFNγ antibody (BD Pharmingen), followed by streptavidin conjugated to alkaline phosphatase (BDPharmingen) and BCIP-NBT substrate (Sigma). Spots were counted using an Immunospot plate reader (v. 5.0; Cellular Technology, Ltd.). Assays of recall response to corneal challenge were similar except that mice were immunized with 4×10^4^ pfu of virus or control supe, and cervical lymph nodes were removed 4 d post-challenge. Dilutions of 2×10^5^ to 6×10^4^ cells from individual mice were added to wells containing gB498–505 peptide, and dilutions of 1×10^6^ to 2×10^5^ cells were added to wells containing UV-inactivated HSV-1.

### *In vitro* assessment of the effects of B7-2 expression

For preparation of dendritic cells (DCs), bone marrow harvested from B7KO mice was differentiated *in vitro* by incubation for 7 d in RPMI containing 10% FCS, 1% L-glutamine, 40 ng/ml recombinant mouse GMCSF and 10 ng/ml recombinant mouse IL-4. Eighteen hr before the end of culture, cells were left uninfected or were infected with Δ41Δ29 or Δ41Δ29B7-2 at moi of 5, washed and returned to culture at 4×10^4^ to 8×10^4^ cells/well in a 96-well flat-bottom plate. Representative wells were analyzed by flow cytometry using Fc block followed by fluorophore-conjugated antibodies to CD3, CD11b, CD11c, MHC class II, and CD86.

For isolation of CD4^+^ T cells, mononuclear cells were isolated from the spleens of DO-11.10 mice [specific for an epitope of chicken ovalbumin (OVA) presented on I-A^d^] using lymphocyte separation medium (ICN). T cells were enriched by negative selection using a Pan T cell isolation kit II (Miltenyi Biotec), according to the manufacturer's instructions. Purity of T cell preparations was verified by flow cytometric analysis of an aliquot using Fc block followed by anti-CD3-APC and anti-CD4-Alexa700, as well as PE-labeled antibodies to CD19, CD11b, CD11c, MHC class II, and CD86. Remaining T cells were cultured (2 to 2.5×10^5^/well) with Δ41Δ29- or Δ41Δ29B7-2-infected DCs or uninfected DCs in medium alone or containing 200 µg/ml OVA. Cultures were incubated for 3 d, and then CD3^+^CD4^+^ T cells were analyzed for forward scatter by flow cytometry.

### IL-2 assay

IL-2 content in 50 µl of T∶DC culture supernatants collected 66 hr after addition of T cells to APCs was assayed on CTLL by standard MTT assay [34]. Briefly, CTLL cells (5×10^3^/well) were washed twice and placed in culture in RPMI+10% FCS, to which was added dilutions of IL-2 (for standard curve) or T cell culture supernatants. After 48 hr of culture MTT substrate was added and 4 hr later absorbance at 490 nm was measured on a BioRad 680 reader.

### Quantitation of serum antibodies

To determine the titer of HSV-specific serum antibodies induced by vaccine, mice were immunized with vaccine virus or control supernatant. Blood was collected from the tail vein of mice 21 d after immunization. Serum was prepared by clot retraction and analyzed by ELISA as previously described [35]. Anti-mouse-IgG-biotin (R & D Systems, Minneapolis, MN) was used as secondary antibody and detected using streptavidin-HRP followed by OPD substrate (Sigma, St. Louis, MO). Alternatively, anti-mouse-IgG1-HRP and -IgG2a-HRP (SouthernBiotech) were used. Plates were read at 490 nm on a BioRad 680 reader. Antibody concentrations were determined by comparison to standard curves generated with serum containing known concentrations of IgG captured on plates coated with goat-anti-kappa light chain antibody (Caltag) as previously described [35].

### Immunization of mice for vaccine efficacy studies

Hind flanks of mice were injected s.c. with 4×10^5^ pfu (high), 4×10^4^ pfu (medium), or 4×10^3^ pfu (low) doses of Δ41Δ29, Δ41Δ29B7-1, Δ41Δ29B7-2 suspended in 40 µl total vol. Cohorts of mice received an equivalent amount of supernatant concentrated from uninfected cell cultures as a negative control for immunization.

### *In vivo* challenge

Four wk after immunization, mice were anesthetized by intraperitoneal injection of ketamine/xylazine, and infected with 5 µl HSV-1 mP inoculated onto each scarified cornea for a dose of 8×10^5^ pfu/mouse. To measure virus replication in the corneal epithelium, eyes were swabbed with moistened cotton-tipped swabs at 4 hr and days 1 through 5 post-infection. Swabs for each mouse were placed together in 1 ml PBS and stored frozen until assay. Virus was quantified on Vero cell monolayers by standard plaque assay. After challenge, signs of disease and survival were monitored on a daily basis. Blepharitis scores were assigned in masked fashion based on the following scale: 0, no apparent signs of disease; 1, mild swelling and erythema of the eyelid; 2, moderate swelling and crusty exudate; 3, periocular lesions and depilation; and 4, extensive lesions and depilation. Mean daily disease score was calculated for each group. Keratitis was assessed at 9 d and 14 d post-challenge using an ophthalmoscope and scored in masked fashion based on the following scale: 0, no apparent signs of disease; 1, mild opacity; 2 moderate opacity with discernible iris features; 3, dense opacity; 4, dense opacity with ulceration. Virus replication in neural tissue was analyzed by dissection of TG and brainstems from a cohort of mice 3 d or 5 d after challenge. Tissues were stored frozen until use. For virus titer determination, tissues were thawed and disrupted using a Mini-Bead Beater (BioSpec, Inc.), and then diluted for standard plaque assay.

### Isolation of T cells in the trigeminal ganglia

Mononuclear cells were isolated from TG of immunized or control BALB/c mice 4 d post-challenge as previously described [36]. Briefly, the 2 TG from each mouse were dissected and pooled (pooling was necessary to isolate sufficient cells from naïve animals). The tissue was minced and incubated for 1 hr at 37°C in a solution of 400 U/ml collagenase type I (Sigma) in DME containing 10% FCS. TG were dissociated by tituration and washed, resuspended in medium, and suspended material was passed through a cell strainer (70 µm). Cells were then treated with Fc block, stained with antibodies to CD3, CD4 and CD8 and analyzed by flow cytometry.

### Assessment of latency by real-time PCR

The 2^(ΔΔCt)^ method [37], [38] was used to compare relative amounts of latent viral DNA in TG after detection by real-time PCR. TG were collected 30 d post-challenge from mice that had received the medium dose of vaccine, and were stored at −80°C. DNA was isolated from the TG using a QIAamp DNA Mini Kit (Qiagen) according to the manufacturer's instructions. PCR reactions were run in 25 µl reaction vol using FastStart SYBR Green Master (Rox) (Roche), and primers at 300 nM final concentration. For GAPDH, reactions used 10 ng template DNA and primers forward 5′-GAGTCTACTGGCGTCTTCACC-3′ and reverse 5′-ACCATGAGCCCTTCCACAATGC-3′ which amplify a 337 bp product. For HSV-1 UL50, reactions used 125 ng template DNA and primers forward 5′-CGGGCACGTATGTGCGTTTGTTGTTTAC-3′ and reverse 5′-TTCCTGGGTTCGGCGGTTGAGTC-3′ which amplify a 195 bp product. Reactions were performed using an ABI Prism 7500 real-time PCR system (Applied Biosystems) and cycle conditions: 2 min at 50°C, 5 min at 95°C, 40 cycles of 95°C for 15 sec and 60°C for 1 min. Specificity was verified by melting curve analysis. The average of duplicate wells yielded the Ct value, and the UL50 signal for each sample was normalized to the GAPDH signal content by determination of ΔCt. Fold decrease in UL50 content of TG from Δ41Δ29B7-1 or Δ41Δ29B7-2-immunized mice relative to mice receiving Δ41Δ29 was determined using the 2^(ΔΔCt)^ method [37], [38].

### Statistics

Significance of difference in virus titers in the cornea on individual days was determined by Student's t test. The Kruskal-Wallis non-parametric test was used to assess the significance of difference in blepharitis scores on individual days post-challenge. Differences in T cell responses, keratitis scores, virus titer in the nervous system, and relative levels of latent viral DNA were compared using one way analysis of variance (ANOVA). Each group immunized with Δ41Δ29 was compared with Δ41Δ29B7-1 or Δ41Δ29B7-2 using the Bonferroni post hoc test.

## Results

### *In vitro* characterization

**Figure 1:**
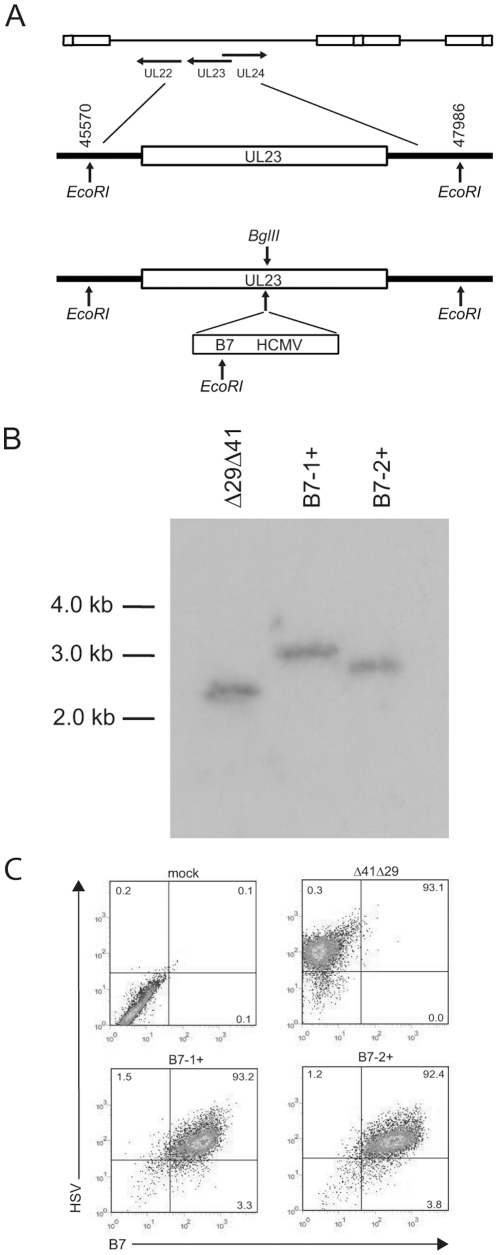
Construction and characterization of B7-expressing viruses. (A) The genomic position of the tk ORF is shown on line 1. An expanded view of this region (line 2) shows the location of EcoRI restriction enzyme sites. Line 3 shows the insertion cassette containing the HCMV IEp fused to either the B7-1 (Δ41Δ29B7-1) or B7-2 (Δ41Δ29B7-2) ORF, each of which contains an EcoRI site near the carboxyl terminus. B) Southern blot analysis of the *tk* locus. Genomic DNAs isolated from the Δ41Δ29 parental and recombinant viruses were digested with EcoRI, subjected to electrophoresis, and transferred to membrane. The blot was hybridized to a ^32^P-labeled fragment of p101086.7 DNA. The expected sizes of the EcoRI fragments were 2416 bp for Δ41Δ29 (lane 1), 3242 bp for B7-1 virus (lane 2), and 2923 bp for B7-2 virus (lane 3). C) B7 molecule expression on the surface of cells infected *in vitro* with Δ41Δ29B7-1 or Δ41Δ29B7-2. S2 cell monolayers were mock-infected or infected with the indicated virus at moi of 5, then collected and stained 24 hr later with rabbit anti-HSV-2 followed by goat anti-rabbit-PE, and with the appropriate anti-B7-biotin antibody followed by streptavidin-FITC. Cells were analyzed by flow cytometry.

To construct B7-expressing viruses, cDNAs encoding murine B7-1 and B7-2 driven by the HCMV IEp were inserted into the HSV-1 thymidine kinase (tk) ORF 751 bp from the 5′ ATG in plasmid p101086.7BglII. The resulting plasmids were cotransfected into S2 cells with full-length DNA from the replication-defective HSV-1 strain Δ41Δ29 [22], which contains a lacZ insertion in the ICP8 ORF and a nonsense linker in the *vhs* ORF (Figure 1A). Plaques were isolated from the cotransfection mixture in the presence of acyclovir because the recombinant viruses are functionally impaired for tk activity. Cells infected with the plaque isolates were screened for expression of B7 molecules by flow cytometry. B7-1- and B7-2-expressing recombinants were triply plaque-purified and named Δ41Δ29B7-1 and Δ41Δ29B7-2, respectively. Southern blot analysis was used to verify insertions into the *tk* locus in Δ41Δ29B7-1 and Δ41Δ29B7-2 (Figure 1B). Genomic DNAs purified from the Δ41Δ29 parental and potential recombinant viruses were restricted with EcoRI, electrophoresed, transferred to membrane, and hybridized to a ^32^P-labeled fragment of p101086.7BglII DNA. The Southern blot of Δ41Δ29 showed a single fragment of expected size (2.4 kb; Figure 1B, lane 1), and single fragments of expected sizes 3.2 kb and 2.9 kb for the B7-1 and B7-2-containing viruses, respectively (Figure 1B, lanes 2 and 3).

Expression of B7 costimulation molecules on the surface of cells infected *in vitro* with Δ41Δ29B7-1 or Δ41Δ29B7-2 was demonstrated by flow cytometry (Figure 1C). S2 cells were mock infected or infected, collected and stained 24 h later with anti-B7-1 and B7-2 antibodies. Mock-infected cells or cells infected with Δ41Δ29 (Figure 1C, left panels) showed no B7 staining above background, whereas cells infected with Δ41Δ29B7-1 or Δ41Δ29B7-2 stained brightly for B7-1 or B7-2, respectively (Figure 1C, right panels). These data indicate host costimulation molecules are uniformly expressed at high levels on the surface of cells infected with Δ41Δ29B7-1 or Δ41Δ29B7-2.

### Immune response to vaccination

**Figure 2:**
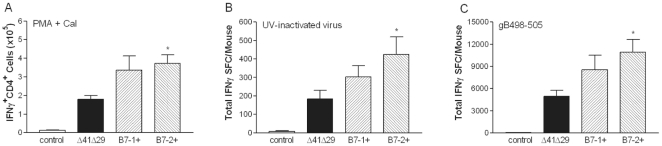
IFNγ-producing T cells induced by immunization. Groups of mice were immunized with 4×10^5^ pfu of the indicated replication-defective virus or control supernatant. Six days after immunization cells from the draining lymph nodes were isolated. A) Lymph node cells from BALB/c mice were stimulated with PMA and CaI, then were stained with antibodies to CD3 and CD4, permeabilized and stained with anti-IFNγ and analyzed by flow cytometry. Data represent the arithmetic mean ± SEM of the absolute number of CD4^+^IFNγ^+^ cells per mouse. B) Lymph node cells from BALB/c mice were stimulated *in vitro* with UV-inactivated HSV-1 and analyzed in an IFNγ ELISpot assay. C) Lymph node cells from BALB.B mice were stimulated *in vitro* with 0.2 µM of peptide representing the CD8 epitope gB498–505 and analyzed in an IFNγ ELISpot assay. Data represent the arithmetic mean ± SEM of the absolute number of IFNγ-producing cells per mouse. Data were compiled from 3 independent experiments for each of panels A, B and C. For each set of 3 experiments the total number of mice used was 4 to 9 mice for the control group, and 11 to 12 mice for each vaccine group. *, P<0.05 for Δ41Δ29 compared with Δ41Δ29B7-2.

The capacity of B7 costimulation molecules expressed by the immunizing virus to elicit cellular and humoral immune responses was determined. To investigate the CD4^+^ T cell response, mice were immunized s.c. with Δ41Δ29B7-1, Δ41Δ29B7-2, the parental ICP8^−^vhs^−^ virus Δ41Δ29, or an equivalent amount of control supernatant. Six days later cells in the draining lymph nodes were stimulated with PMA and CaI and analyzed by intracellular staining for IFNγ. All of the vaccine viruses elicited a greater number of CD4^+^ T cells producing IFNγ than did control supernatant (Figure 2A; P<0.0001 by ANOVA) but significantly, immunization with Δ41Δ29B7-2 stimulated greater expansion of IFNγ-producing, CD4^+^ T cells than did immunization with Δ41Δ29 (Figure 2A). Because bystander activation of CD4^+^ T cells can occur in response to HSV infection [39]–[41], additional mice were immunized and cells from the draining lymph nodes were isolated 6 d later and cultured with UV-inactivated HSV-1 in an IFNγ ELISpot assay. Although the number of HSV-specific, cells producing IFNγ was lower after UV-HSV-1 stimulation than when cells had been stimulated with PMA and CaI, immunization with Δ41Δ29B7-2 again stimulated greater expansion of IFNγ-producing, CD4^+^ T cells than did immunization with Δ41Δ29 (Figure 2B). Δ41Δ29B7-1 also appeared to stimulate more IFNγ-producing CD4^+^ and CD8^+^ T cells than Δ41Δ29, though the result was not statistically significant (P<0.1).

To investigate HSV-specific CD8^+^ T cell response to the vaccine viruses, cells from the draining lymph nodes of mice immunized as described above were cultured with peptide representing the immunodominant CD8^+^ T cell epitope gB498–505 presented by H-2K^b^ [32], [33], [42], and were analyzed by IFNγ ELISpot. Immunization of mice with Δ41Δ29, Δ41Δ29B7-1 and Δ41Δ29B7-2 all stimulated greater expansion of HSV epitope-specific, IFNγ-secreting CD8^+^ T cells than did control supernatant (Figure 2C; P<0.0001 by ANOVA). Significantly more CD8^+^ T cells specific for HSV were found in mice immunized with Δ41Δ29B7-2 than with Δ41Δ29 (Figure 2C). This observation was corroborated by analysis using tetramer staining of CD8^+^ T cells specific for the gB498–505 epitope (data not shown). Δ41Δ29B7-1 also appeared to stimulate more IFNγ-producing CD4^+^ and CD8^+^ T cells than Δ41Δ29, though the result was not statistically significant (P<0.1). The above data expressed as a proportion of lymph node cells producing IFNγ yielded similar results (Figure S1). Collectively, these assays indicate stronger induction of HSV-specific T cell responses by replication-defective viruses that express costimulation molecules, particularly B7-2.

Stimulation of naïve CD4^+^ T cells requires contact with antigen-containing cells expressing both MHC class II and B7 costimulation molecules. To verify that MHC class II^+^ cells can express virus-encoded B7 molecules, DCs generated from the bone marrow of B7KO mice were infected with Δ41Δ29 or Δ41Δ29B7-2. MHC class II^+^ DCs infected with Δ41Δ29 did not express B7-2 (Figure S2A, left panel). In contrast, B7-2 was detected on nearly half of the MHC class II^+^ DCs infected with Δ41Δ29B7-2 (Figure S2A, right panel). To determine whether virus-encoded B7-2 expression had a functional consequence, CD4^+^ T cells enriched from splenocytes of DO11.10 mice were incubated with OVA and infected B7KO DCs. The DCs infected with Δ41Δ29B7-2 and incubated with OVA induced more pronounced CD4^+^ T cell blastogenesis than DCs infected with Δ41Δ29 and incubated with OVA (Figure S2B). Δ41Δ29B7-2-infected DCs also stimulated more IL-2 production by the DO-11.10 T cells (Figure S2C).

**Figure 3:**
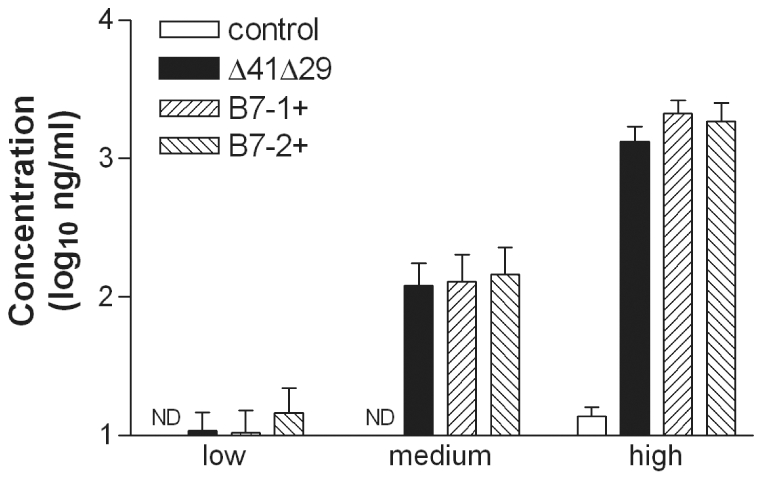
Prechallenge HSV-1-specific serum IgG titers. Groups of BALB/c mice were immunized with low, medium or high doses of the indicated viruses. Blood was collected 21 d after immunization and concentration of HSV-specific IgG in serum was determined by ELISA. Data represent the geometric mean ± SEM compiled from 2 independent experiments for a total of 10 to 12 mice per group for each dose. ND, not detectable (OD≤wells containing PBS).

The capacity of the vaccines to elicit HSV-specific antibody was determined by immunizing groups of mice s.c. with control supernatant or 4×10^5^ pfu (high), 4×10^4^ pfu (medium) or 4×10^3^ pfu (low) doses of Δ41Δ29, Δ41Δ29B7-1 or Δ41Δ29B7-2. Three wk after immunization, blood was collected and HSV-specific IgG in the sera was quantified by ELISA. HSV-specific serum antibody was generated by immunization with all of the virus strains, but at no dose were the antibody responses elicited by Δ41Δ29B7-1 or Δ41Δ29B7-2 significantly greater than those induced by Δ41Δ29 (Figure 3). Subsequent experiments, however, did show a modestly higher concentration of HSV-specific IgG in BALB/c or BALB.B mice immunized with the medium dose of Δ41Δ29B7-2 virus compared with Δ41Δ29 (data not shown). The ratio of HSV-specific IgG1 to IgG2a was approximately 1∶1 in all vaccine groups in all experiments (data not shown), indicating the character of the humoral response was not altered by B7 expression. Thus, the moderately stronger T cell responses induced by virus expressing costimulation molecules generally did not manifest as significant additional help for antibody production.

### Protective effect of the vaccines

**Figure 4:**
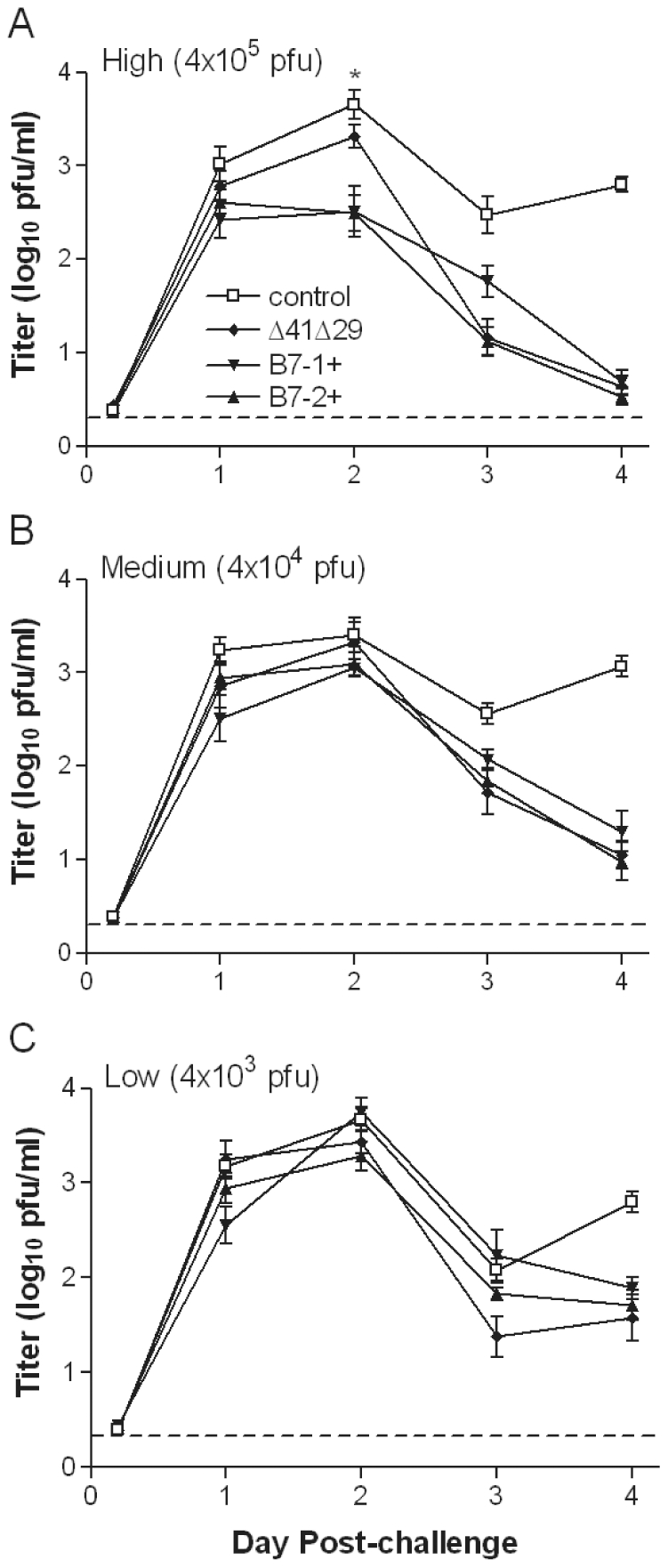
Titer of challenge virus shed from the corneal epithelium. Groups of BALB/c mice were immunized with A) high, B) medium or C) low doses of the indicated virus or control supernatant. All groups were challenged 1 mo after immunization by inoculation of HSV-1 mP onto the corneas and mouse eyes were swabbed at the indicated times post-challenge. Titers of virus collected on swabs were determined by standard plaque assay. Data represent the geometric mean ± SEM for 10 to 12 samples per group at each dose, compiled from 2 independent experiments. *, P = 0.002 to 0.014 for Δ41Δ29 compared with Δ41Δ29B7-1 and Δ41Δ29B7-2. Dashed line indicates limit of detection in the plaque assay.

Many vaccine formulations protect when given to mice at high doses and/or in multiple doses. We chose a more challenging test of efficacy by giving a single vaccination with relatively low virus doses. Groups of BALB/c mice were immunized with 4×10^5^ pfu (high), 4×10^4^ pfu (medium) or 4×10^3^ pfu (low) doses of virus or with control supernatant. At 4 wk after immunization mice were challenged on the corneas with the virulent HSV-1 strain mP. Replication in the corneal epithelium was quantified over the first 4 d post-challenge by titration of virus collected on corneal swabs. Mice immunized with control supernatant experienced high levels of challenge virus replication in the corneal epithelium (Figure 4). All three vaccine strains reduced the amount of virus replicating in the corneal epithelium; the high, medium or low doses of vaccine reduced virus titers below the level observed with control supernatant by 2, 3, or 4 d post-challenge, respectively. Interestingly, immunization with the B7-1 or B7-2-expressing viruses did not decrease virus infection in the cornea compared with Δ41Δ29 except in the high dose immunization group (Figure 4A). Even at the high dose, the effects of B7-1 and B7-2 expression were transient.

**Figure 5:**
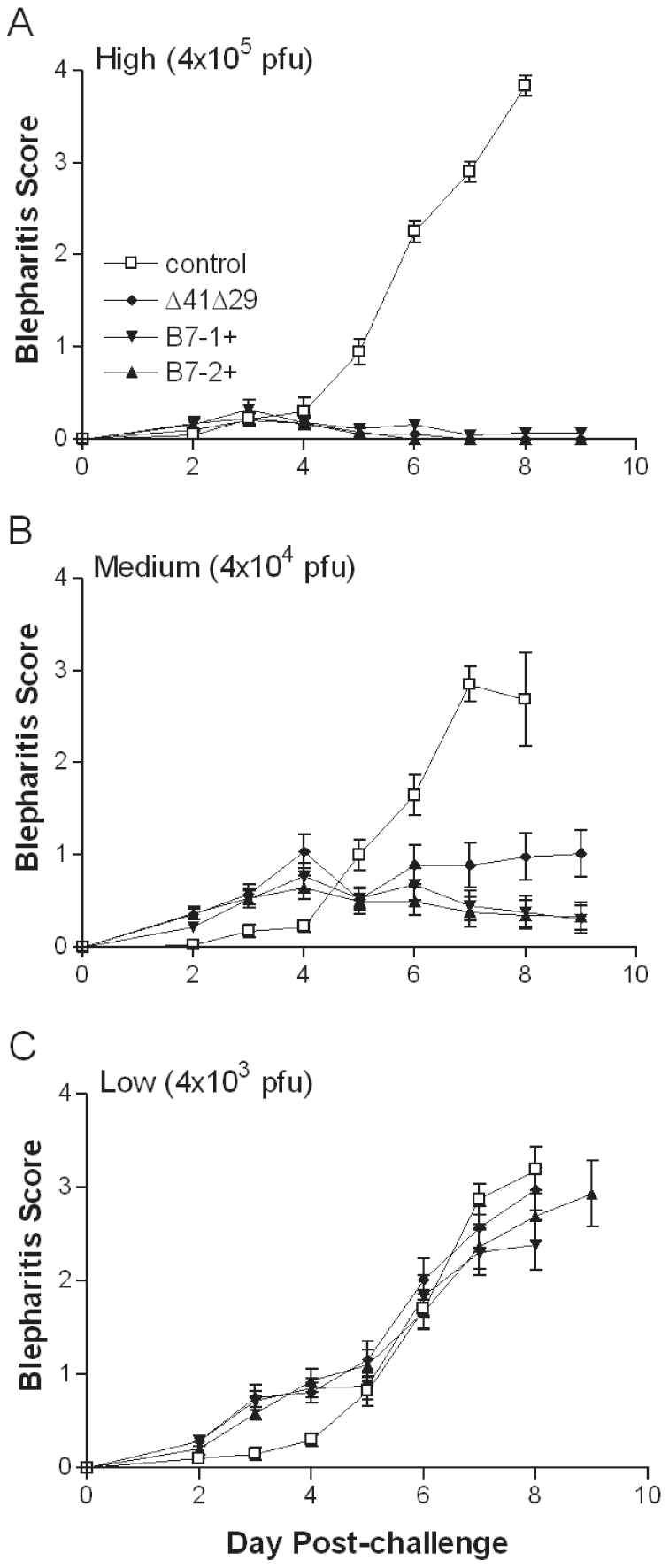
Severity of blepharitis post-challenge. Mice were immunized with the A) high, B) medium, or C) low doses of the indicated virus or control supernatant and challenged as described in Figure 4. Blepharitis was scored daily after challenge in masked fashion. Data represent the mean ± SEM for all mice compiled from 2 independent experiments (total = 20 eyes for control, and 24 to 30 eyes for each group of virus-immunized mice at each dose).

Blepharitis developed in mice immunized with control supernatant by 4 d post-challenge, and became severe by 7 d post-challenge (Figure 5). In marked contrast, all 3 replication-defective vaccine strains protected mice very effectively from eyelid inflammation when given at the high dose (Figure 5A). Δ41Δ29B7-1 and Δ41Δ29B7-2 still protected mice almost completely from blephariits at the medium dose, though protection afforded by Δ41Δ29 appeared to wane slightly (Figure 5B). The lowest vaccine dose did not protect mice from blepharitis (Figure 5C). Thus, all vaccine strains provided protection against blepharitis at the high and medium doses, but viruses encoding either B7-1 or B7-2 did not significantly enhance this protection over that afforded by Δ41Δ29.

**Figure 6:**
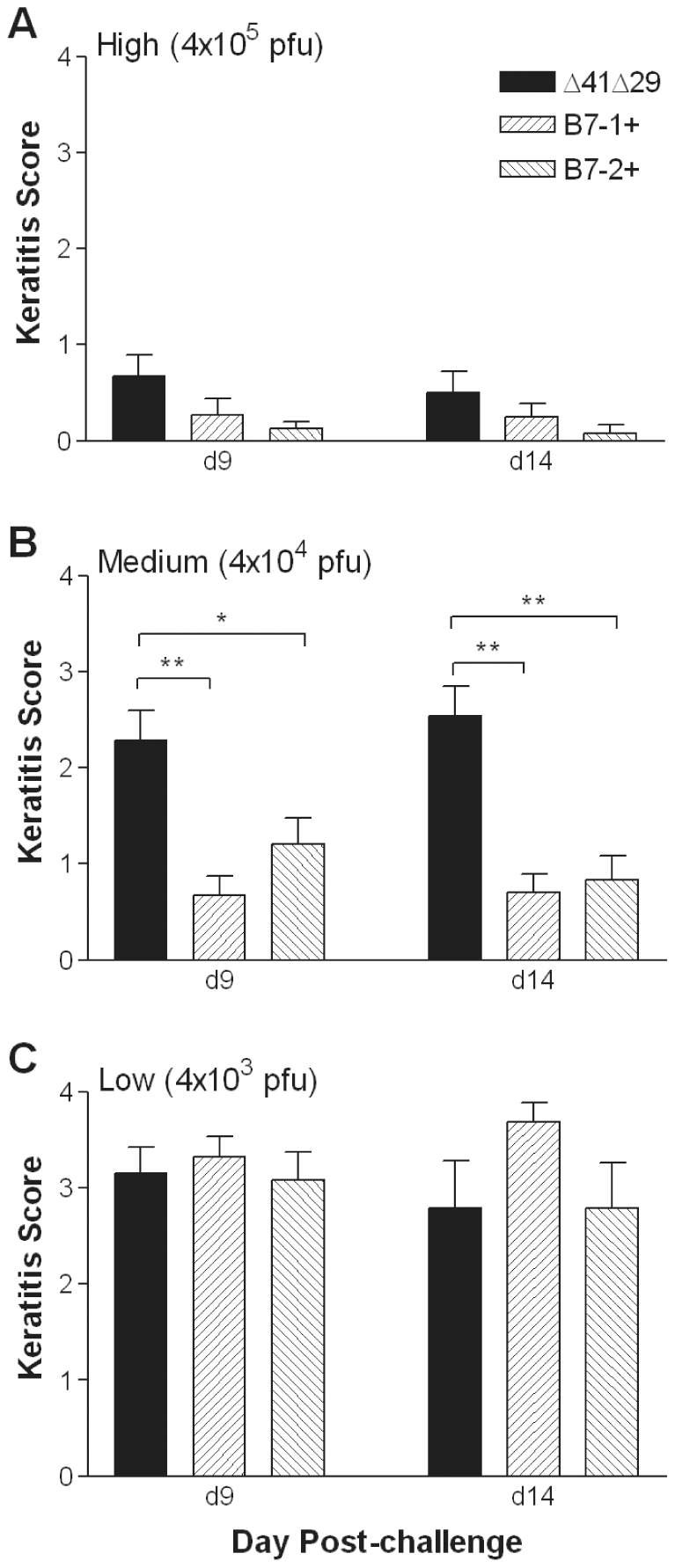
Incidence of keratitis. Eyes of mice were scored in masked fashion for signs of keratitis 9 d and 14 d post-challenge. Mean scores are shown for groups immunized with the A) high, B) medium, or C) low doses of the indicated virus or control supernatant. Data represent the mean ± SEM for all mice surviving on the indicated day and were compiled from 2 independent experiments (total = 22 to 24 eyes for each high dose group on days 9 and 14; 28 to 30 eyes for each medium dose group on days 9 and 14; 20 to 26 eyes for each low dose group on day 9 and 14 to 16 eyes for each low dose group on day 14). **, P<0.001; *, P = 0.01 for Δ41Δ29 compared with Δ41Δ29B7-1 and Δ41Δ29B7-2.

Keratitis was assessed in surviving mice at 9 and 14 d post-challenge. Most mice immunized with control supernatant did not survive to day 9. In contrast, all mice receiving high or medium doses of any of the vaccine viruses survived, as did most mice receiving low dose vaccine, so these mice were evaluated for keratitis. Each of the three vaccine strains given at the high dose protected mice almost completely from keratitis at 9 d post-challenge (Figure 6A), and no mouse immunized with the high dose of Δ41Δ29B7-2 showed more than mild disease. At the medium dose, most mice immunized with Δ41Δ29 showed moderately severe corneal disease after HSV-1 infection. In contrast, disease was mild in mice previously immunized with the medium dose of either B7-1 or B7-2-expressing virus (Figure 6B), and many corneas were clear. At the lowest dose of vaccine, the B7-expressing vaccine strains did not protect the mice from developing keratitis better than Δ41Δ29 (Figure 6C). Results at 14 d post-challenge were very similar (Figure 6), although by this time some of the mice in the low dose immunization groups had died. Thus, immunizations using the medium dose (4×10^4^ pfu) revealed that B7 molecules encoded by the vaccine virus significantly enhance protection against corneal disease over that afforded by the ICP8^−^vhs^−^ parental virus.

**Figure 7 pone-0022772-g007:**
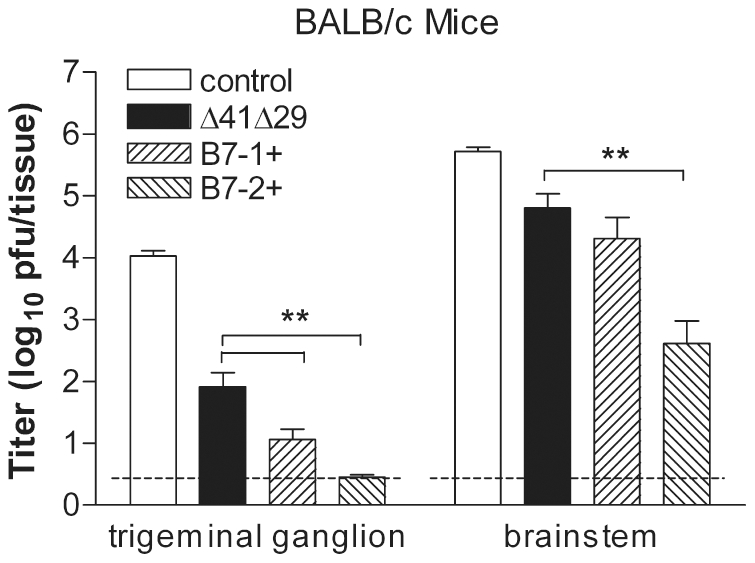
Acute replication of challenge virus in the nervous system. BALB/c mice were immunized with the medium dose of the indicated virus or with control supernatant and challenged by the corneal route one month later. TG and brainstems were dissected 5 d post-challenge, and virus titer in them was determined by standard plaque assay. Data represent the geometric mean ± SEM for a total of 20 TG and 10 brainstem samples per group, compiled from 2 independent experiments with similar results. **, P<0.001 compared with Δ41Δ29. Dashed line indicates limit of detection in the plaque assay. (P<0.001 for all virus groups compared with control supernatant for TG; P<0.01 to 0.001 for B7-expressing viruses compared with control supernatant for brainstem).

HSV-1 reaches the TG in as little as 2 d after corneal infection unless impeded by pre-existing immunity [43]–[46]. We therefore determined whether protection from keratitis afforded by immunization with Δ41Δ29B7-1 and Δ41Δ29B7-2 viruses was related to the level of challenge virus in the nervous system. Mice immunized with the medium dose were chosen for analysis because this dose had permitted the best distinction between vaccine strains based on corneal disease. To assess acute infection of the nervous system, groups of BALB/c mice were challenged by corneal infection with HSV-1 4 wk after immunization, and TG and brainstems were isolated 5 d post-challenge for determination of virus titer. All vaccine strains protected the nervous system compared with control supernatant. Importantly, both Δ41Δ29B7-1 and Δ41Δ29B7-2 vaccination reduced challenge virus replication in the TG better than Δ41Δ29 (Figure 7). Encephalitis, though rare in humans, can be devastating and thus it is important to know whether the central nervous system is protected through vaccination. In the brainstem, Δ41Δ29B7-2 significantly reduced challenge virus replication compared with Δ41Δ29, and both B7-expressing viruses but not Δ41Δ29 had a significant protective effect compared with control supernatant (Figure 7).

**Figure 8:**
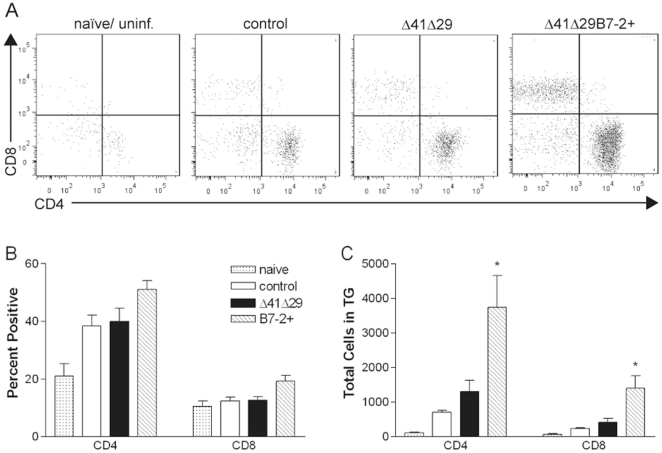
T cells in the TG in response to challenge. BALB/c mice were immunized with control supernatant or the medium dose of Δ41Δ29 or Δ41Δ29B7-2 and challenged with HSV-1 by the corneal route one month later. TG were dissected 4 d post-challenge, pooled for each mouse, and digested with collagenase to release mononuclear cells. CD3^+^ T cells isolated from the TG were analyzed for coexpression of CD4 or CD8 by flow cytometry. A) Representative scatter plots of T cells in the dissociated TG of mice from the indicated immunization groups. B) Percentage of CD3^+^ T cells costaining with anti-CD4 or anti-CD8. C) Total CD4^+^ and CD8^+^ T cells recovered from the dissociated TG of each mouse. Data were compiled from 2 independent experiments, with a total number of 4 naïve mice and 6 mice in each immunization group. *, P<0.05 to 0.01 for Δ41Δ29 compared with Δ41Δ29B7-2.

To determine whether the recall T cell response was associated with decreased challenge virus replication in the nervous system, we examined T cells in the TG and cervical lymph nodes after challenge. Groups of BALB/c mice were immunized, subsequently infected via the cornea as described above, and mononuclear cells infiltrating the TG were isolated 4 d post-challenge. Corneal infection caused an influx of primarily CD4^+^ T cells into the TG of immunized mice compared with naïve controls (Figure 8A and B, P<0.05 to 0.001). The frequency of CD8^+^ T cells increased only in the TG of mice previously immunized with Δ41Δ29B7-2 (Figure 8A and B, P<0.05 compared with naïve controls). Significantly, previous immunization with Δ41Δ29B7-2 permitted a greater total number of CD4^+^ and CD8^+^ T cells to infiltrate the TG compared to previous immunization with Δ41Δ29 (Figure 8C). Thus, an increase in T cell infiltration temporally coincided with lower virus titers in the TG acutely after challenge of mice immunized with Δ41Δ29B7-2.

To determine whether virus-specific T cells were recalled to the ocular region by challenge virus infection, we analyzed HSV-specific T cell responses in the draining (cervical) lymph nodes 4 d after corneal challenge of BALB.B mice. HSV-specific CD4^+^ T cells producing IFNγ were present in greater numbers in the local lymph nodes of previously immunized mice, and a much larger frequency (Figure S3A) and absolute number (Figure S3B) of CD4^+^ T cells was found in mice immunized with Δ41Δ29B7-2 compared with Δ41Δ29. All previously immunized mice had a greater frequency and absolute number of HSV-specific, IFNγ-producing CD8^+^ T cells in the cervical lymph nodes than did mice immunized with control supernatant, but there was no detectable difference among vaccine strains (Figure S3C and D). Because these analyses were performed in BALB.B mice to monitor gB498–505 epitope-specific CD8^+^ T cells, we verified a corresponding reduction in virus titer in the TG and brainstems of the BALB.B mice immunized with B7-expressing virus (Figure S4). Thus the nervous system was better protected by immunization with Δ41Δ29B7-2 than with Δ41Δ29 when assessed at either 4 d or 5 d post-challenge in BALB.B or BALB/c mice.

**Figure 9:**
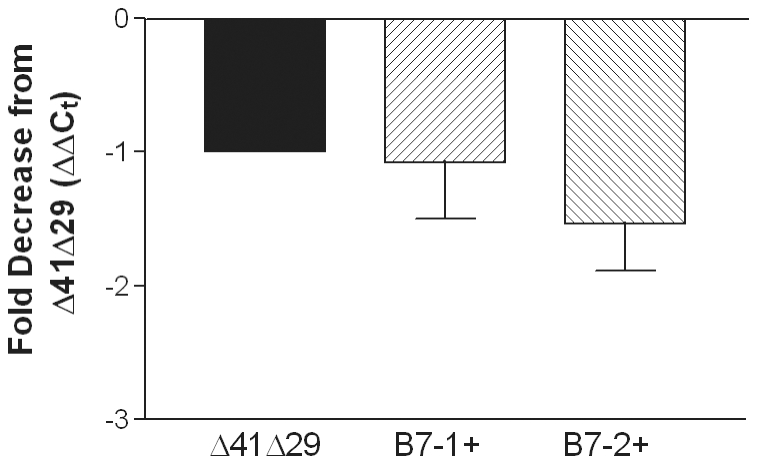
Relative levels of HSV-1 DNA in trigeminal ganglia during latency. Groups of mice immunized with the medium dose of virus were challenged with 8×10^5^ pfu HSV-1 mP 1 month later. Four weeks after challenge, TG were removed and DNA was extracted. Relative viral DNA content was assessed by real-time PCR using primers for UL50 after normalization to the signal for GAPDH. Data represent the relative mean fold decrease (±SD) of latent genome in 11 TG from Δ41Δ29B7-1- and 11 TG from Δ41Δ29B7-2-immunized mice compared with 11 TG from Δ41Δ29-immunized mice (set to 1). P>0.05 by ANOVA.

The capacity of vaccination to reduce establishment of latency was determined by detection of challenge virus DNA in the TG. Groups of mice were immunized with the medium dose of vaccine viruses and subsequently infected on the cornea with HSV-1. DNA was purified from individual TG 30 d post-challenge, and their burden of challenge virus genome was determined by real-time PCR using primers for UL50 to detect viral genomes and for GAPDH as a normalization control. UL50 results for each mouse were normalized to GAPDH content and the relative amounts of UL50 were then compared between groups. The reduction in genome load in mice immunized with either of the B7-expressing viruses compared with Δ41Δ29 was not statistically significant (Figure 9). The genome load in one control-immunized mouse that survived challenge was 6 to 8-fold greater than that in mice immunized with any of the vaccine strains (data not shown), suggesting that all three viruses do afford some protection from latent infection of the nervous system.

## Discussion

Although long-term antiviral therapy of persons with milder HSV keratitis has slightly reduced the clinical impact of HSK, a vaccine is still highly desirable to prevent HSK and obviate the need for, and side-effects and expense of, such antiviral therapy. A viable HSV vaccine candidate must meet goals of both safety and efficacy. Replication-defective virus vaccines address these goals because they do not reproduce and spread in the recipient, and they stimulate broad spectrum immune responses due to the numerous viral proteins expressed in infected cells. Further manipulation of prototype replication-defective viruses has enhanced their immunogenicity and effectiveness. Using replication-defective virus, the current approach of combining vhs deletion with virus-encoded expression of host costimulation molecules has achieved the best protection yet against HSV-1-induced keratitis in a mouse model. The increased efficacy of Δ41Δ29B7-2 correlates with enhanced virus-specific CD4^+^ and CD8^+^ T cell responses. Δ41Δ29B7-2 also protects the peripheral and central nervous systems from acute infection significantly better than the Δ41Δ29 virus lacking B7. Thus, combining vhs deletion with provision of costimulation signals encoded from the replication-defective virus genome further enhances T cell-mediated immune protection, specifically against HSV-1-mediated corneal disease and neurological infection.

The dose at which vaccines are tested can reveal their power to protect under the most challenging conditions. Many vaccine formulations are efficacious when given multiple times or at high doses. Indeed, all of the vaccine strains tested herein protect effectively against blepharitis and keratitis at an immunizing dose of 4×10^5^ pfu. A single vaccination with just 4×10^4^ pfu of cell free Δ41Δ29B7-1 or Δ41Δ29B7-2 revealed significantly enhanced protection against HSK compared with Δ41Δ29. Δ41Δ29B7-2 also protected better than Δ41Δ29 against acute infection of the nervous system. Thus, two important goals of prophylactic vaccination to reduce or prevent disease were achieved using a modest dose of B7-expressing, ICP8^−^vhs^−^ virus. Protection was lost for all vaccine strains when given at 4×10^3^ pfu, but this is not surprising considering the extremely low dose.

Deletion of vhs and expression of B7-2 likely make unique contributions to the efficacy of Δ41Δ29B7-2. The vhs deletion may contribute more significantly to generation of HSV-specific antibody: Immunization with Δ41Δ29 elicits a stronger antibody response than its replication-defective parent [22], whereas B7-2 encoded by replication-defective (ICP8^−^) HSV-2 [25] or ICP8^−^vhs^−^ HSV-1 (Figure 3) does not as readily enhance the antibody response. Virus-encoded B7-2, on the other hand, contributes significantly to T cell activation after immunization with either HSV-2 or HSV-1 B7-expressing strains (Figures 2, S2 and [25]). Interestingly, these observations indicate that the increased T cell activation does not primarily manifest itself as additional help for antibody production in mice that express endogenous B7 costimulation molecules. Instead, protection against disease and nervous system infection seen after challenge of mice immunized with ICP8^−^vhs^−^ HSV-1 or ICP8^−^ HSV-2 strains encoding B7 costimulation molecules correlates with the increased numbers of IFNγ-producing T cells these B7-expressing viruses elicit (Figures 6 through [Fig pone-0022772-g007] 8 and [25]). Thus, while deletion of a viral immune evasion molecule from replication-defective HSV-1 improves vaccine efficacy [22], virally encoded B7 molecules make an additional contribution to vaccine-mediated protection against HSK.

The impact of vaccines encoding B7 costimulation molecules, or any replication-defective vaccine, on initial replication of challenge virus is more modest. Reduced replication at the site of inoculation typically occurs beginning 2 to 4 d post-challenge depending on immunization dose (Figure 4 and [22]), possibly because virus-immune T cells must be recalled from a resting state before their activity becomes apparent. Nonetheless, HSV-1 strains expressing B7 costimulation molecules clearly reduce corneal disease compared with their ICP8^−^vhs^−^ parent (Figure 6). Mott et al. [47] also observed that the amount of virus replication in the corneal epithelium of mice undergoing primary infection did not predict the incidence or severity of corneal disease. Presumably, higher doses of vaccine virus would reduce replication of virus in the corneal epithelium, as previously observed [22].

The mechanism underlying improved protection of the cornea against HSK and the nervous system against infection is not known but likely involves the Δ41Δ29B7-2-induced T cell response. We observed an inverse correlation between virus titers in the TG and T cell infiltration at 5 d post-challenge (Figures 7 and 8). CD4^+^ T cell responses in the draining lymph node were also enhanced just prior to tissue infiltration. Interestingly, CD8^+^ as well as CD4^+^ T cell numbers increased in the TG, as has been previously observed [48], [49], but unlike previous reports only the CD4^+^ T cell response was enhanced in the draining lymph node. This may represent a difference between the recall response and response to acute infection. Alternatively, CD8^+^ T cells may increase in the draining lymph nodes at later times post-challenge [48]. In mice immunized with Δ41Δ29B7-1, the recall T cell response was not quite enhanced to a statistically significant extent relative to Δ41Δ29 but still may be sufficient to protect against HSK better than Δ41Δ29. It must be noted that virus-specific CD8^+^ T cells also play a dominant role in suppressing HSV-1 reactivation in the TG [50], [51], though a role for vaccine-induced CD8^+^ T cells in inhibiting establishment of latency has not been demonstrated [16], [51].

HSK results from the response of antigen non-specific CD8^+^ and Th1 CD4^+^ T cells activated during HSV infection of the cornea of HSV-naïve mice [39]–[41], [48]. Importantly we observed protection of the cornea rather immunopathology in mice with pre-existing antiviral T cell responses elicited with ICP8^−^vhs^−^B7^+^ vaccine. Such protection has been noted with other replication-compromised HSV or lipopeptide vaccines that elicit Th1-like antiviral immune responses [7], [9], [10], [14], [22]. Thus, pre-existing Th1 T cells induced by vaccination that can be quickly recalled to the cornea and TG after virus challenge must proffer a unique advantage. These IFNγ-producing, CD4^+^ T cells may help protect against HSK directly through the antiviral effects of the IFNγ they produce [52], or indirectly by limiting virus infection and inflammation in the corneal stroma and TG, by providing an environment rich in cytokines that reduce bystander activation, and/or by supporting the differentiation of HSV-specific CD8^+^ T cells in response to virus vaccine [53]. A detailed analysis of T cells and cytokines in the corneal stroma and TG acutely after challenge of naïve versus vaccinated mice may reveal the basis for immune-mediated protection against HSK. Regardless of the mechanism, this protective effect has key implications for HSV-1 vaccine design because preservation of a sensitive tissue such as the cornea is crucial to vaccine success.

Δ41Δ29B7-1 and Δ41Δ29B7-2 protect equivalently against keratitis, but B7-2-expressing virus stimulates greater expansion of T cells and provides better subsequent protection from acute infection of the nervous system by HSV-1. The critical signals mediated by B7-1 and B7-2 operate at different temporal phases of T cell activation in response to infection. B7-2 is constitutively expressed and rapidly upregulated for interaction with its cognate receptor [54], whereas B7-1 expression on professional APCs must be provoked. Despite this difference, VSV infection generates equivalent levels of virus-specific CTL and antibody in mice lacking either B7-1 or B7-2 [55], and immunization of B7KO mice with HSV-2 expressing either B7-1 or B7-2 affords equivalent protection against HSV-2 challenge [24]. Our result that Δ41Δ29B7-2 is overall superior to Δ41Δ29B7-1 is intriguing because B7-1 and B7-2 encoded by the vaccine viruses would be expressed with equivalent kinetics. Perhaps in a context where endogenous costimulation molecules are expressed, virus-encoded B7-2 more effectively augments endogenous B7-2 signals to induce stronger T cell activation.

Precedent exists for the beneficial activity of virus-encoded B7 costimulation molecules as a strategic element of vaccines. B7-1 and B7-2 encoded by vaccinia, adenovirus or HSV vectors markedly augment immunogenicity of coexpressed tumor antigens [56]–[58], and help reduce tumor burden in animal models [57], [59]–[61]. HSV strains encoding B7 costimulation molecules represent a new direction in that they enhance the immune response to the pathogen itself. Most viruses, including HSV, infect a variety of cells other than professional APCs. Non-hematopoetic cells are capable of processing and presenting viral antigen and conceivably virus-encoded B7 costimulation molecules confer on these cells the capacity to activate naïve T cells, thus amplifying a response that may otherwise be limited by the inability of replication-defective virus to spread. Noninfectious HSV particles engineered to contain B7 costimulation molecules on their surface also induce stronger immune responses than particles that lack B7 [62], lending further support to the idea that B7 costimulation can be provided exogenously in conjunction with virus antigens to artificially create a professional APC [56], [63], [64]. Whatever the mechanism, addition of B7 expression to ICP8^−^vhs^−^ HSV-1 confers significant protection against HSK with a single dose of just 4×10^4^ pfu. Such capacity to influence disease course in mice using very low doses of vaccine is critical as one envisions scaling up to a vaccine dose that may be protective in humans.

## Supporting Information

Figure S1 Proportion of IFNγ-producing T cells induced by immunization. Lymph node cells depicted in Figure 2 were also analyzed based on A) percentage of CD4^+^ cells stimulated with PMA and CaI that express IFNγ; B) IFNγ SFC per 10^6^ lymph node cells of BALB/c mice stimulated *in vitro* with UV-inactivated HSV-1; and C) IFNγ SFC per 10^6^ lymph node cells of BALB.B mice stimulated *in vitro* with 0.2 µM peptide gB498–505. Data in B and C represent the arithmetic mean ± SEM per 10^6^ lymph node cells per mouse.
(TIF)

Figure S2 Virus-expressed B7 can create antigen-presenting cells. Bone marrow cells from B7KO mice were differentiated *in vitro* using recombinant mouse GMCSF and IL-4. A) CD11c^+^ DCs were analyzed by flow cytometry for MHC class II and B7-2 expression 18 hr after infection with Δ41Δ29 (left panel) or Δ41Δ29B7-2 (right panel). B) DO-11.10 T cells were incubated for 3 d with OVA and Δ41Δ29-infected DCs (unshaded histogram) or Δ41Δ29B7-2-infected DCs (shaded histogram) before analysis of cell size (forward scatter of CD3^+^CD4^+^ T cells) by flow cytometry. C) IL-2 produced in cultures containing DO-11.10 T cells, OVA, and Δ41Δ29-infected DCs or Δ41Δ29B7-2-infected DCs. A representative experiment is shown out of 3 performed. *, P = 0.0271.
(TIF)

Figure S3 IFNγ-producing T cells responding to challenge. Groups of BALB.B mice were immunized with the medium dose of the indicated replication-defective virus or control supernatant. One month later mice were challenged by infected via the cornea with HSV-1. Four days post-challenge, mononuclear cells from the cervical lymph nodes were stimulated *in vitro* with A and B) UV-inactivated HSV-1, or C and D) 0.2 µM of gB498–505 peptide and analyzed in an IFNγ ELISpot assay. Data were compiled from 3 independent experiments with UV-inactivated virus stimulus for a total number of 8 to 10 mice per group. Data were compiled from 4 independent experiments with peptide stimulus for a total number of 12 to 14 mice per group. *, P<0.05 to 0.01 for Δ41Δ29 compared with Δ41Δ29B7-2.
(TIF)

Figure S4 Acute replication of challenge virus in the nervous system of BALB.B mice. BALB.B mice were immunized with the medium dose of the indicated virus or with control supernatant and challenged by the corneal route one month later. TG and brainstems were dissected 4 d post-challenge, and virus titer in them was determined by standard plaque assay. Data represent the geometric mean ± SEM for 12 TG and 6 brainstem samples per group, compiled from 2 independent experiments with similar results. **, P<0.001; *, P<0.01 compared with Δ41Δ29. Dashed line indicates limit of detection in the plaque assay. (For TG, P<0.01 to 0.001 for all virus groups compared with control supernatant; for brainstem, P<0.001 for Δ41Δ29B7-2 compared with control supernatant).
(TIF)
