# Supplementary figures and images for: B7 Costimulation Molecules Encoded by Replication-Defective, vhs-Deficient HSV-1 Improve Vaccine-Induced Protection against Corneal Disease
Source: PLoS One. 2011 Aug 3;6(8):e22772. doi: 10.1371/journal.pone.0022772 (PMC3149624; doi:10.1371/journal.pone.0022772)

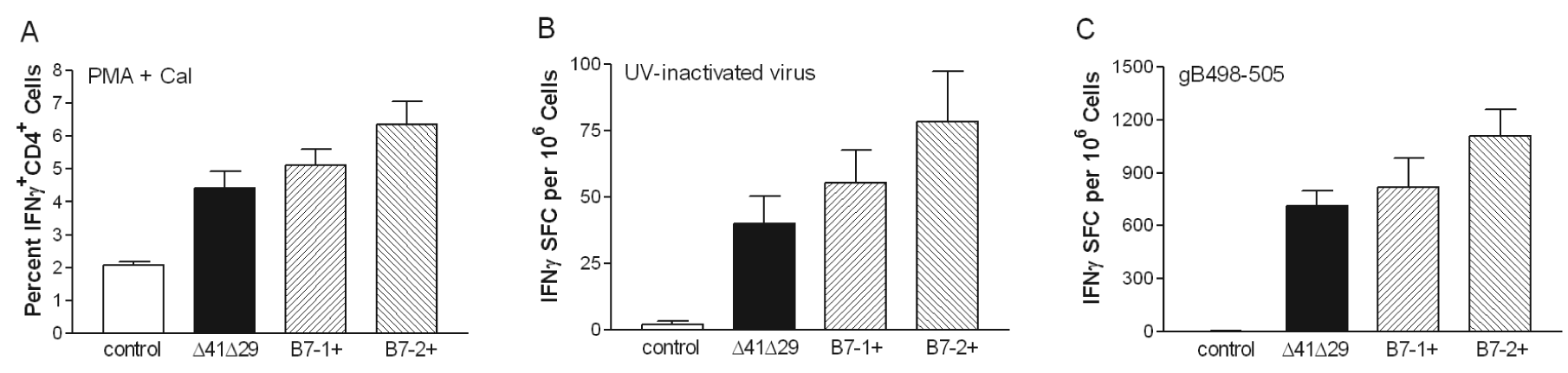

Supplement: Figure S1 — Proportion of IFNγ-producing T cells induced by immunization. Lymph node cells depicted in Figure 2 were also analyzed based on A) percentage of CD4+ cells stimulated with PMA and CaI that express IFNγ; B) IFNγ SFC per 106 lymph node cells of BALB/c mice stimulated in vitro with UV-inactivated HSV-1; and C) IFNγ SFC per 106 lymph node cells of BALB.B mice stimulated in vitro with 0.2 µM peptide gB498–505. Data in B and C represent the arithmetic mean ± SEM per 106 lymph node cells per mouse. (TIF) [file pone.0022772.s001.tif]

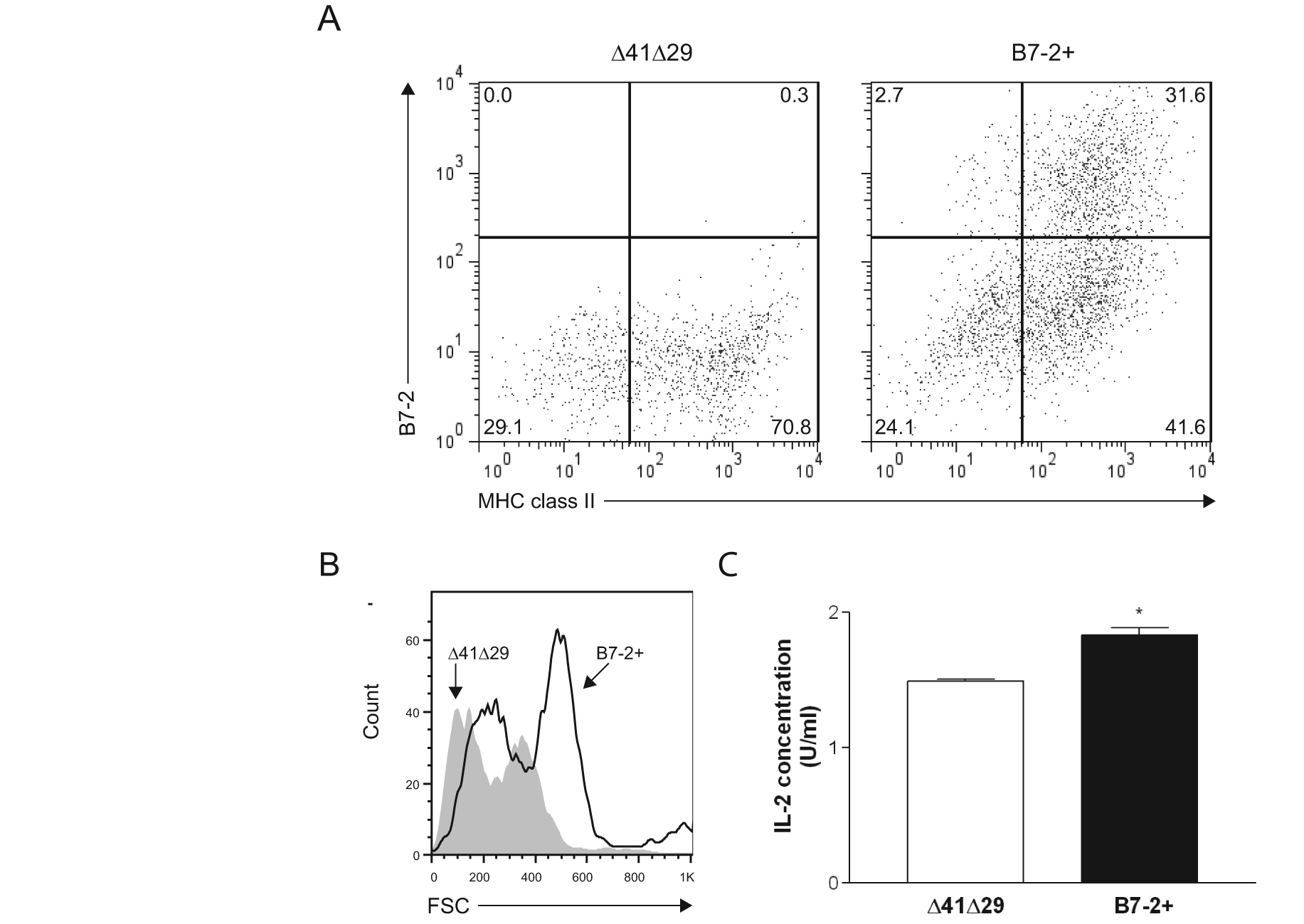

Supplement: Figure S2 — Virus-expressed B7 can create antigen-presenting cells. Bone marrow cells from B7KO mice were differentiated in vitro using recombinant mouse GMCSF and IL-4. A) CD11c+ DCs were analyzed by flow cytometry for MHC class II and B7-2 expression 18 hr after infection with Δ41Δ29 (left panel) or Δ41Δ29B7-2 (right panel). B) DO-11.10 T cells were incubated for 3 d with OVA and Δ41Δ29-infected DCs (unshaded histogram) or Δ41Δ29B7-2-infected DCs (shaded histogram) before analysis of cell size (forward scatter of CD3+CD4+ T cells) by flow cytometry. C) IL-2 produced in cultures containing DO-11.10 T cells, OVA, and Δ41Δ29-infected DCs or Δ41Δ29B7-2-infected DCs. A representative experiment is shown out of 3 performed. *, P = 0.0271. (TIF) [file pone.0022772.s002.tif]

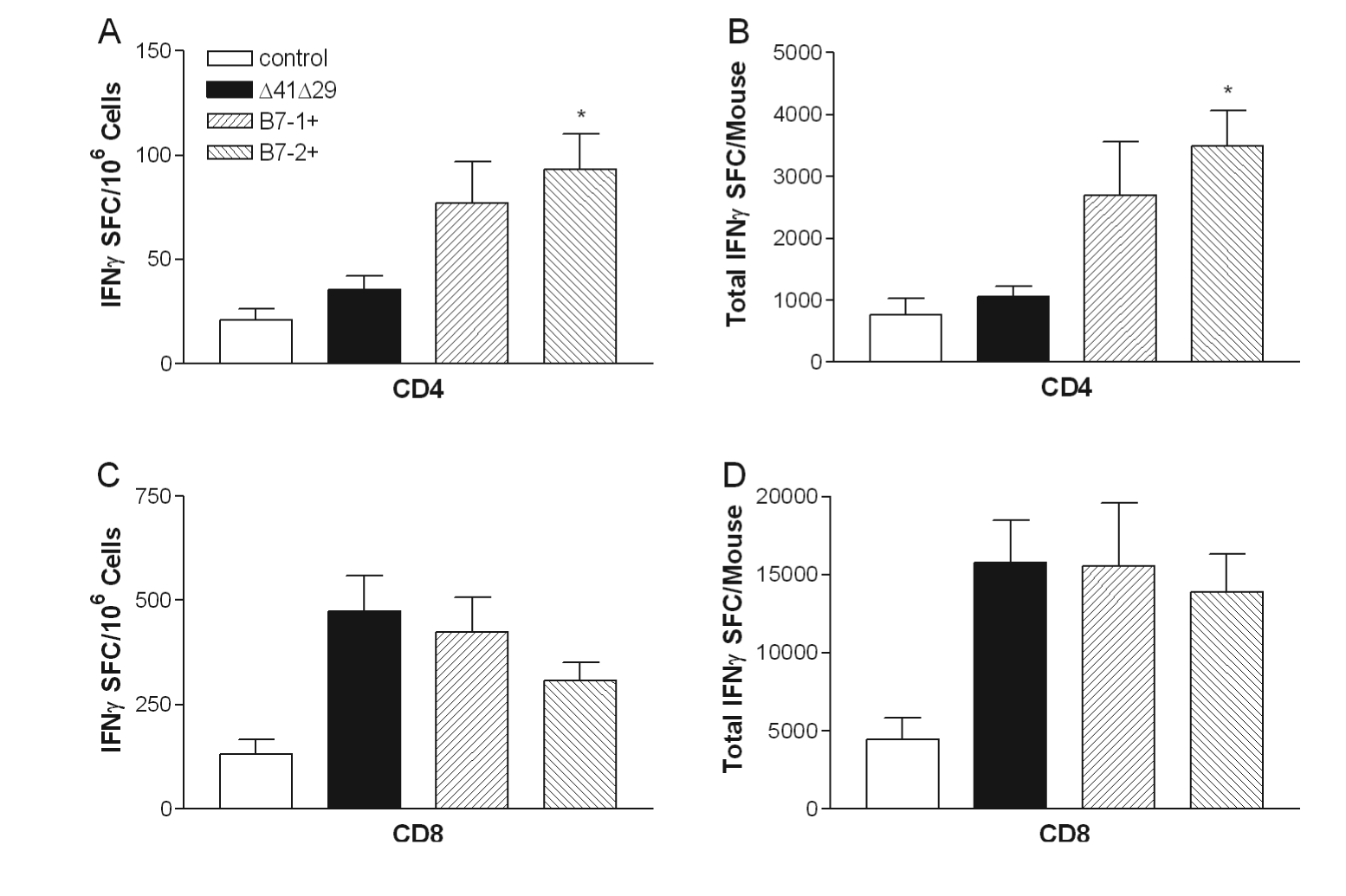

Supplement: Figure S3 — IFNγ-producing T cells responding to challenge. Groups of BALB.B mice were immunized with the medium dose of the indicated replication-defective virus or control supernatant. One month later mice were challenged by infected via the cornea with HSV-1. Four days post-challenge, mononuclear cells from the cervical lymph nodes were stimulated in vitro with A and B) UV-inactivated HSV-1, or C and D) 0.2 µM of gB498–505 peptide and analyzed in an IFNγ ELISpot assay. Data were compiled from 3 independent experiments with UV-inactivated virus stimulus for a total number of 8 to 10 mice per group. Data were compiled from 4 independent experiments with peptide stimulus for a total number of 12 to 14 mice per group. *, P<0.05 to 0.01 for Δ41Δ29 compared with Δ41Δ29B7-2. (TIF) [file pone.0022772.s003.tif]

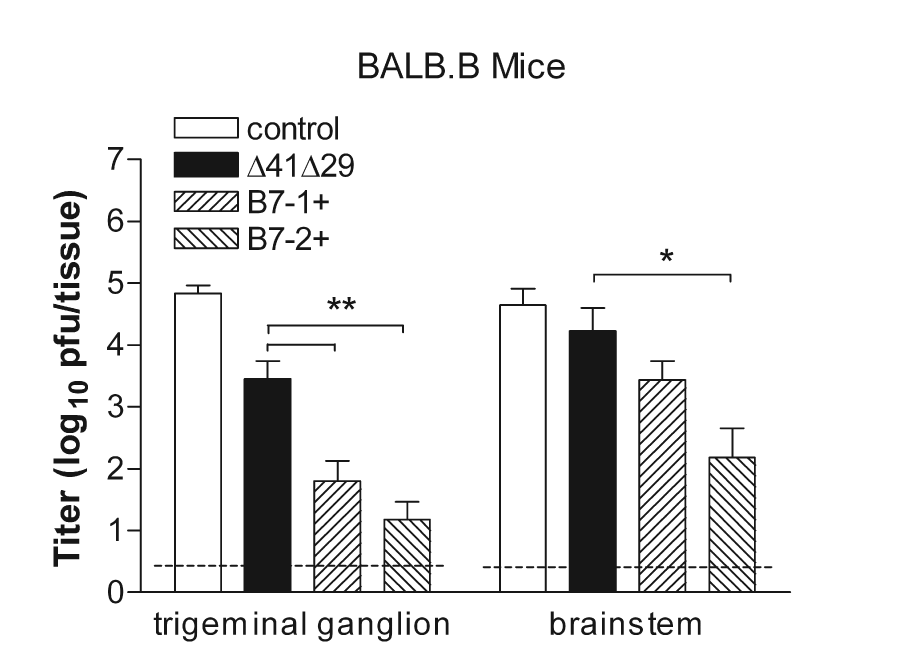

Supplement: Figure S4 — Acute replication of challenge virus in the nervous system of BALB.B mice. BALB.B mice were immunized with the medium dose of the indicated virus or with control supernatant and challenged by the corneal route one month later. TG and brainstems were dissected 4 d post-challenge, and virus titer in them was determined by standard plaque assay. Data represent the geometric mean ± SEM for 12 TG and 6 brainstem samples per group, compiled from 2 independent experiments with similar results. **, P<0.001; *, P<0.01 compared with Δ41Δ29. Dashed line indicates limit of detection in the plaque assay. (For TG, P<0.01 to 0.001 for all virus groups compared with control supernatant; for brainstem, P<0.001 for Δ41Δ29B7-2 compared with control supernatant). (TIF) [file pone.0022772.s004.tif]
